# Sharing linked data sets for research: results from a deliberative public engagement event in British Columbia, Canada

**DOI:** 10.23889/ijpds.v4i1.1103

**Published:** 2019-05-07

**Authors:** Jack Teng, Colene Bentley, Michael M Burgess, Kieran C O’Doherty, Kimberlyn M McGrail

**Affiliations:** 1 Population Data BC, University of British Columbia, 201-2206 East Mall, Vancouver, BC, V6T 1Z3 Canada; 2 Canadian Centre for Applied Research in Cancer Control, BC Cancer, 675 West 10th Ave., Vancouver, BC, V5Z1L3, Canada; 3 W. Maurice Young Centre for Applied Ethics, School of Population and Public Health, Medical Genetics, Southern Medical Program, University of British Columbia, Kelowna, BC, V1V1V7, Canada; 4 Department of Psychology, University of Guelph, MacKinnon Ext (Bldg. 154), 87 Trent Lane, Guelph, ON, N1G2W1, Canada; 5 Centre for Health Services and Policy Research, School of Population and Public Health, University of British Columbia, 201-2206 East Mall, Vancouver, BC, V6T 1Z3 Canada

## Abstract

**Introduction:**

Research using linked data sets can lead to new insights and discoveries that positively impact society. However, the use of linked data raises concerns relating to illegitimate use, privacy, and security (e.g., identity theft, marginalization of some groups). It is increasingly recognized that the public needs to be consulted to develop data access systems that consider both the potential benefits and risks of research. Indeed, there are examples of data sharing projects being derailed because of backlash in the absence of adequate consultation. (e.g., care.data in the UK).

**Objectives and methods:**

This paper describes the results of a public deliberation event held in April 2018 in Vancouver, British Columbia. The purpose of this event was to develop informed and civic-minded public advice regarding the use and the sharing of linked data for research with a focus on the processes and regulations employed to release data. The event brought together 23 members of the public over two weekends.

**Results:**

Participants developed and voted on 19 policy-relevant statements. Voting results and the rationale behind any disagreements are reported here. Taken together, these statements provide a broad view of public support and concerns regarding the use of linked data sets for research and offer guidance on measures that can be taken to improve the trustworthiness of policies and process around data sharing and use.

**Conclusions:**

Generally, participants were supportive of research using linked data because of the value they provide to society. Participants expressed a desire to see the data access request process made more efficient to facilitate more research, as long as there are adequate protections in place around security and privacy of the data.

## Introduction

Research using large and often complex linked data sets can lead to new insights that inform policy, service delivery, the efficiency of processes and finances, and/or the distribution of resources [1,2]. Researchers seek more access to linked data sets, including data from new sources and types of data, such as patient-reported information, genomic information, data from wearable devices, and social media. Multiple, linked data sets are increasingly available to and used by both public and private organizations [3]. The increasing digitization of data, technical advances in linking complex data sets, and scientific advances in analyzing the resulting data all contribute to this trend. Current policies and practices around the sharing of linked data will need to adapt to reflect who can have access to these expanded resources, under what circumstances and for what purposes [4].

One tension inherent in the use of linked data for research is that there is both the potential to benefit society and some level of risk to privacy, security, and of unethical behaviour toward individuals represented in the data [5]. As the Nuffield Council on Bioethics notes, the “public interest” includes both supporting responsible use of data and protecting the privacy of individuals [6], and recent public engagements suggest the public does in fact hold quite nuanced views on data sharing [7,8]. These engagements illustrate that the public is not automatically for or against issues such as sharing of data or the involvement of the private sector, but instead focuses on the context, including considerations such as who is asking, for what, to be used for what purpose [9,10].

Building on this base, further research is needed to understand how underlying values translate to potential trade-offs, such as (but not exclusive to) between research and privacy, and how risks can be mitigated. Including the public in development of these policies is consistent with a recent consensus statement on principles of public engagement for data-intensive research [11]. The importance of doing so is highlighted in cases where the perception of inadequate consultation resulted in data sharing projects being derailed (e.g., care.data in the UK) [12,13]. Resulting policies can ultimately be informed by both expert knowledge and public perspectives and interests. “Expert” is used here to identify individuals who are professionals and academics working in data-related fields, whose perspectives are shaped by those interests and relations. Members of the public are also “experts” who bring important knowledge from their varied experience and from the work of considering their values in the context of practical decisions in complex contexts; it is this latter expertise that is made manifest in meaningful engagement.

This paper describes a public deliberation event that took place in April 2018 and the policy-relevant statements that emerged from that event. This deliberation was designed to identify public perspectives and interests on sharing linked data for research. It focused on the processes and regulations determining the sharing of linked data for research (e.g., data access rules, data steward practices, researcher data use). For the purposes of this study, the processes and regulations that were examined were those practiced by data stewards in BC, Canada (i.e., designated individuals in public organizations responsible for deciding on whether data can be shared). A well-established approach to deliberative public engagement was implemented for the study [14]. The deliberation was titled Using Data About You for Research: Who, How, and Why, and focused on information governance, and more specifically the issues surrounding the use and sharing of linked data sets for research purposes. The event was led by Population Data BC [15], in collaboration with researchers at the University of British Columbia, the University of Guelph, the University of Edinburgh, and with partners from across BC.

Participants formulated and voted on 19 policy-relevant statements which we report here as the collective output of the deliberation. The statements covered the following topics: the governance of linked data sets; the security and review process for releasing linked data sets; the responsibilities of data stewards and researchers; and the involvement of the public. This paper describes the methods of recruitment, the deliberation approach used, the conclusions reached by the deliberative forum, and their implications for future research and practice.

## Methods

### Public deliberation

Public deliberation events are informed by political theory on deliberative democracy and recognition that it is important to have citizen input on social issues that are controversial or a source of concern [16]. The main premise of a public deliberation is that while they may have differences in values, opinions, and interests, members of society need to find common rules and practices with which all can live. Public deliberation is based on the recognition that members of society can make important contributions to public policy based on the diversity of life experiences they bring to bear on a given topic.

The purpose of public deliberations is for participants to deliberate among themselves and reach collective statements or policy recommendations that accommodate their varied perspectives. While reaching consensus on the statements or recommendations is encouraged, if it is not attained, the reasons for the disagreements are documented. They therefore offer important insights both on the issues on which a group of citizens can agree, and also on more thorny areas on which there is persistent disagreement [14]. As such, public deliberations can produce outputs that, if followed, can enhance the democratic legitimacy of programs, actions, and decisions [17].

Public deliberations can be distinguished from public consultations and other forms of research (e.g., surveys, focus groups) by the depth and length of the discussions, the amount of relevant information provided to and by participants, and how the participants themselves create the recommendations that are conveyed to policy makers [18,19]. Public consultations often collect participants’ views, whereas deliberations are intended to create collective statements that reflect how participants think their diverse interests are best accommodated [20].

### Participant recruitment

The aim of the recruitment was to assemble a group of British Columbia residents who reflect a diverse set of experiences and interests. In this respect, they constituted a mini-public, which represents “the diversity of social characteristics and the plurality of initial points of view in the larger society” [16]. To allow for a public deliberation, a mini-public should avoid vested or sectarian interests that can be influenced by specific political directions and thus undermine trust in the deliberative process [21].

A market research company was used to facilitate the recruitment, as they have developed lists of public members who have agreed to participate in marketing research and community engagement events for research. This approach to recruitment does have the potential bias that it selects for participants who are already interested in these types of events, which is a form of self-selection that may under-represent the opinions of public members who are not prone to participating in survey and other research. This is a challenge that is shared with all recruitment approaches, which we attempted to mitigate through stratification for under-represented groups in deliberative forums, and by covering expenses and paying participants for their time, as described below.

Potential participants were first sent an online letter of invitation by the market research company to attend the deliberation. The dates of the event, the honorarium ($150/day), and a commitment to cover travel and accommodation costs were specified in the letter. To register their interest in participating, potential participants were asked to provide demographic information so that respondents could be stratified by age, sex, geography, size of their community, ethnicity, income, and education. The marketing company screened the participants based on our demographic requirements and provided the lists of suitable participants. The research team then contacted potential participants by phone and managed the final stages of the recruitment process. The demographic breakdown of the participants was developed based on the recruitment of previous public deliberations in an attempt to achieve demographic diversity (i.e., using census statistics as a basis and then over-representing specific groups) [22]. People who work as privacy professionals were excluded because their vested interest and potential expert status might inhibit deliberation. Previous research has shown that lay members are more hesitant to offer their views and feel empowered to participate when the deliberating group includes experts on the particular topic area of the deliberation [20].

Geography was included as a factor in recruitment because it might impact attitudes toward and/or the level to which people are exposed to the use of personal data. We took care to have representation from remote communities, as determined by community size and distance from an urban centre (which in Canada can be large – the variable is called the Metropolitan Influence Zone [23]). We were conscious of recruiting participants from all age groups and made special attempts to include participants aged 18-24 years, as previous deliberations indicated this group is difficult both to recruit and to retain [24]. This age group is thought to have a higher familiarity with technology use and may thus have a different relationship to data from those with low technology use. We made a specific effort to recruit individuals who identified as Indigenous, as there are distinct norms and practices around data and data sharing in Indigenous communities that were important to reflect in the deliberations [25]. These recruitment choices were made to ensure “normic diversity,” where the selection of participants is meant to ensure the presence of non-majoritarian voices as input to decisions that complements dominant voices and experts [26].

### Deliberation content and participant preparation

**Figure 1: Timeline of public deliberation event and preparations:**
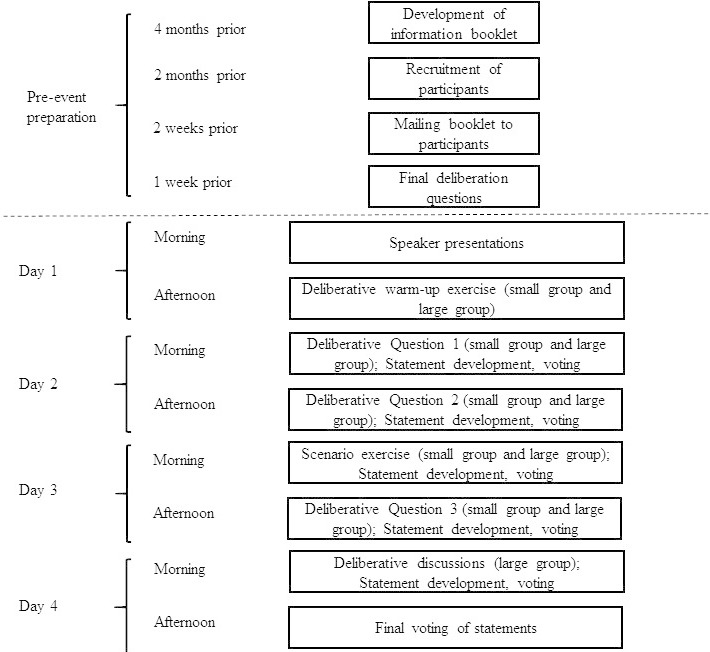


The deliberation was structured around three questions and an exercise using plausible data access request scenarios. See Figure 1 for a breakdown of the timeline of pre-event preparations and the event activities.

The deliberation questions and the scenarios were developed by the research team prior to the event. Participants were asked to provide feedback on the scenarios and to indicate any concerns about them and what changes they would recommend. The following questions and tasks guided the deliberation event:

1. What is important information to consider when approving access to and use of linked data?
2. When is it justified to grant access to linked data, and what measures are important to reduce risks?
3. Working with scenarios: applying previous discussions to work out trade-offs and recommendations
4. What processes would make the assessments of risks and benefits from the use of linked data trustworthy?

Participants were not required to have prior knowledge about the use of linked data for research. Prior to and during the event, information was provided that offered a broad range of views on the issues central to the deliberation. This supported individuals to participate confidently in the discussions, to feel comfortable expressing their views, and to be able to engage with and respond to other participants’ contributions [17].

An information booklet developed by the research team was mailed to participants two weeks in advance of the event. The booklet described what linked data sets are, how they are collected and created, what regulations need to be followed to share them, and current issues and concerns surrounding their use. The booklet was written at a Grade 10 reading level and was reviewed for accuracy by experts [27].

On the first day of the event, the participants heard presentations from five speakers representing a range of perspectives, including:

1. Researcher: how linked data sets can be used to address pressing issues, and the steps and length of time it takes to obtain the data;
2. Data steward: the challenges in deciding on data access requests, both in terms of interpreting regulations and legislation and working with other data stewards;
3. Privacy advocate: the risks and dangers of sharing linked data sets with regards to data security and the maintenance of privacy;
4. Patient: the benefits of research from linked data sets, and the health impacts of having difficulty accessing and sharing their own health data with health providers; and,
5. Indigenous community member: the negative impacts that research can have on identifiable and vulnerable populations.

Speakers were all stakeholders, either by merit of being experts in their field (e.g., data steward, researcher) or as advocates for particular perspectives (privacy advocate, patient, Indigenous community member). The premise underlying our presentation of information in general, and selection of speakers in particular, was that when it comes to ethical considerations, there are no truly “neutral” positions. Rather, there are competing values, interests, and perspectives. Our knowledge-user colleagues helped to identify what was important to include in the booklet as well as identify speakers. While we insisted that the information presented by speakers was factually accurate, we did not ask them to present their information in a neutral manner. Rather, we asked them to present the information from their particular perspective and to be explicit about the reasoning behind their position (this approach has been termed “framing for deliberation” by Friedman, 2007) [28]. The purpose of this framing was to make potentially competing interests explicit to participants of the public deliberation, so that these tensions could be discussed and worked through in the deliberative process.

Participants had the opportunity to ask questions immediately after the presentations, during the deliberation, and at a panel discussion where all the speakers answered questions. Some presenters observed, but none participated in the deliberations, as mixing lay and expert voices can potentially marginalize the views of non-experts [29]. After the first day, the research team answered new questions that emerged or provided additional information when it was requested. The participants asked for further explanation about the types of data research being conducted, aspects of data security (e.g., deidentification), and the review process for data access requests.

### Deliberative process

The deliberation event occurred over two non-consecutive weekends (i.e., a total of four days) in April 2018 and was run by facilitators trained specifically for this type of public deliberation [30]. The large group facilitator was a member of the research team, while the four small group facilitators were current or previous graduate students with experience in facilitation. The time between the weekends gave participants the opportunity to return home, reflect on the discussions, and discuss the topic with their family and friends. All proceedings were audio recorded and transcribed.

Participants met in small groups (four groups with six to eight participants each) and large group (all participants) sessions during the event. One small group and one large group session was devoted to each of the four deliberation questions. The intent of the small group sessions was to encourage participation by all attendees, as well as generate a broad range of viewpoints on each deliberative question. Subsequently, when participants convened in the large group, they worked towards finding common ground that could be translated into statements on the use and sharing of linked data for research.

Whenever discussions began to converge in the large group setting, the facilitator helped participants formulate preliminary statements. Participants then worked together to edit each statement until it represented a collective position that might be viewed as a policy statement. Participants voted on the statement (yes (Y), no (N), or abstain (A)) and after vote counts were asked for their reasoning. Statements, voting, and reasoning behind votes were collectively explored and documented in the large group setting.

The final day was used to review and summarize the group’s statements. This was an opportunity for participants to change their vote in light of new information and changing perspectives. The day concluded with a panel discussion with experts with data- and policy-related responsibilities who heard and discussed the participants’ statements and answered participant questions.

## Results

Of the 31 participants invited to attend, 28 British Columbians initially participated in the deliberation. The number of participants in the final stage of the event decreased to 23 as some participants withdrew owing to personal circumstances (e.g., health, emergency scheduling issues). Though we did have good representation of people aged 18-24 years, we were able to recruit but not maintain representation from Indigenous participants (one Métis-identified participant remained). The reasons provided by the participants for leaving were sickness and life circumstances (e.g., requiring to work on short notice). Supplementary Appendix 1 describes the participant characteristics.

Participants developed and then voted (yes, no, or abstain) on 19 statements. We use the term “statements” as opposed to “policy recommendations” because voting revealed that not all were universally supported; that is, despite the attempt to craft statements that represented consensus views, when it came to voting there often were remaining disagreements. These ranged from issues with wording to persistent disagreements about content. The research team noted that in some cases participants were reluctant to vote “yes” to a statement with which they agreed, because they understood it already to be in place. Specific instances of these complexities are highlighted below.

The participants’ statements are grouped into four categories: 1. the governance of linked data sets; 2. the security and review process for releasing linked data sets; 3. the responsibilities of data stewards and researchers; and 4. the involvement of the public. We describe these areas in broad terms. Within each category, the statements below are ordered by the degree of agreement that the participants reached on them. Further details on voting are available in a separate summary report [31]. It is important to note that the focus of this analysis is to report the statements and their support by the participants as a group [21]. The objective of deliberation is to have the group of participants articulate statements that reflect their collective view of how to make decisions while considering the diversity of views that they have considered, or the “civic-minded” advice on “how to live together.” The statements are, as much as possible, in the words that were negotiated by the full group, and distinct from the analytic or emergent themes characteristic of most qualitative analysis[32]. For this kind of analysis, we therefore do not present quotes from the deliberation, as these would be reflections of individual participants’ views, not the collective statements that were the product of the dialogical act of deliberation. Audio-recording, notetaking and transcription support the accuracy of our reporting of participants’ collectively negotiated statements, and will be used in more depth in future papers.

### 1. The governance of linked data sets

Overall, there was general support that linked data should be available for research, given certain contexts and conditions. This general feeling was most emphasized when discussing the potential for fast-tracking data access requests, particularly in urgent cases such as the public health emergency (explicitly declared in British Columbia) around opioid overdoses [33]. Participants were concerned about delays unnecessarily creating barriers to important research, and expressed that governance should be efficient. Participants often referred to the speakers who indicated that it can take a long time for data stewards and researchers to secure approvals for access to linked data sets.

This general support was tempered with some concerns that increased speed in the approval process might mean cutting corners, resulting in superficial data access reviews. Participants also questioned whether there would be consistency in rules for access and data protection across organizations, and whether efficiency and protecting privacy would be at cross-purposes. Disagreements on increasing efficiency revolved around a desire for more specifics on logistical details, and a feeling that a specific fast-track was not necessary as there was an impression that policy-makers would already have that discretion.

**Table:** 

Statements and recommendations – governance of linked data sets	Y	N	A

1. Develop a plan to make the data linkage approval process more efficient, without compromising the evaluation process.	19	0	4
2. It is important to invest in a collection of linked data sets to promote efficient research while enhancing privacy protection.	16	2	4
3. Policy makers should establish categories that identify requests that require different paths or speed for review, e.g., fast-track for urgent research priorities.	14	8	1
4. If a commercial entity funds research with linked data, it should not be involved in the production and review of that research.	9	0	14
5. There should be a committee or governing body with authority to: Provide oversight and investigation for breaches and/or harms Apply penalties or other consequences Develop policies to mitigate the potential for future breaches and/or harms Intervene when data stewards disagree Develop and operate an appeals process Provide certification for data stewards	8	7	8

Throughout discussions there was continuing emphasis on the role of data stewards and concern over a lack of overall guidelines and regulations governing access to linked data. Participants discussed the potential for disagreements among data stewards on data sharing policies and expressed concern that there was no clear mechanism to resolve those disagreements. Participants began discussion of the creation of a committee to oversee data stewards’ activities and practices. This discussion did not reach a consensus on the committee’s specific responsibilities. There was also concern about the potential for the process to become politicized and proliferate committees, thereby undermining important research.

Participants desired clarity on what the involvement of commercial entities would entail. They were concerned about commercial entities being involved in research and had reservations about the profit motive would affect the use of linked data and the research results by commercial entities, but this discussion did not produce a recommendation. A desire among some participants for commercial entities not to have direct access to data conflicted with others’ opinions that if corporations contributed money to a research project they should have some benefit from the results of the research. There was general agreement that it was inevitable that commercial entities would use data, so a recommendation prohibiting this would not be useful.

Some participants were concerned that centralizing linked data could increase security risks. They were also concerned that a centralized database would result in a single person being in charge of making decisions about access with a potential for errors or excessive influence. Participants frequently expressed interest in having some mechanism of enforcement and consequences if any improper data sharing or usage should occur but this did not result in a recommendation.

### 2. Security and review process for releasing linked data sets

Participants expressed a desire for the security of data sets, including mechanisms to ensure that the assessment of the scientific merit of research proposals is conducted by qualified parties. They also desired appropriate protections for data during the research process. Discussions of these issues led to statements and recommendations on the importance of third-party review of research proposals, best practice guidelines for data storage and access, and review of resulting research results.

Participants were concerned about the risk that research may have harmful impacts on the populations studied, particularly in the case of vulnerable and marginalized populations (e.g., children, Indigenous communities). They recognized that harms may result unintentionally, perhaps even without the researchers’ knowledge. This discussion resulted in the statements about an independent ethics review for all linked data requests, not just those identified as “research” (statement 9). Participants who abstained to the statement did so because they felt there was currently an ethics review in place. Those who abstained were also concerned about introducing another procedure that might slow down the data access request.

Participants did not see the need for an additional independent party to review research results in relation to the original intent or for an independent assessment of the necessity of the requested data for the proposed research (statements 10, 11). The review of research resulting from linked data was seen as under the purview of the peer review process for publications. Those who voted against the statement felt the independent party would be redundant, might lack enforcement power, and/or could impinge on academic freedom. Participants commented on the importance of the independence of research activities, and acknowledged that the direction of research is not predictable, and that what researchers do is sometimes by necessity different from what they propose.

**Table:** 

Statements and recommendations – Security and review process for releasing linked data sets	Y	N	A

6. Scientific review of the research proposal should be performed by an independent party.	23	0	0
7. There should be best practices and guidelines for secure storage and access to linked data.	23	0	0
8. Results and publications of linked data research must be reviewed to ensure that they are justified by the analysis of the data.	21	2	0
9. The proposed research and data access should be reviewed by an independent ethics committee to ensure benefits outweigh potential harms (e.g. potential for re-identification, stigma).	15	0	8
10. Research results should be reviewed by a qualified independent party to reaffirm the original purpose of the research.	6	17	0
11. An independent party should assess requests for data to be sure that the data are necessary to conduct the research.	4	7	12

**Table:** 

Statements and recommendations – Data steward responsibilities	Y	N	A

12. Data stewards should have standard training or certification to ensure appropriate expertise for their role.	19	0	4
13. Data stewards should have standard policies and procedures to guide their work and there should be a certifying body to maintain them.	18	4	1
14. Research using linked data must be monitored by data stewards to ensure data are used in accordance with the original request.	8	13	2

### 3. The responsibilities of data stewards and researchers

#### Data steward responsibilities

The role of data stewards was specifically considered, noting (based on information provided) that data stewards do not have standardized training to do their role, resulting in variations in the way in which data access requests are processed. Participants were concerned this would decrease the efficiency of the data access request process, and suggested processes to standardize data stewards’ practices (e.g., certification). Abstentions reflected concern that a formal certification process would be too cumbersome, and whether it was feasible to standardize data stewards’ practices across different organizations.

Participants proposed that a certifying body could develop and maintain common policies and practices for data stewards (statement 13). The participant who abstained felt that the individual institutions should be responsible for adhering to appropriate policies and procedures and should not require a certifying body. Some participants who were against a certifying body explained that the responsibilities of a certifying body were unclear, while others were generally against certification bodies.

There was no consensus on whether data stewards should monitor whether linked data are being used in accordance with the original request. Those who disagreed with monitoring thought it was an inefficient use of the data stewards’ time and were concerned this may result in an overreach of their responsibilities. Those who abstained were concerned that the task of monitoring research would not be feasible for data stewards due to resource limitations.

#### Researcher responsibilities

In the context of the potential implications of research on vulnerable populations, participants emphasized the responsibility of researchers to conduct studies in an ethical manner, without harming the populations they study.

Participants wished to ensure that those gaining access to linked data are adequately informed of confidentiality requirements and restrictions on the dissemination of data. They also recommended that there should be agreements in place between the researcher and the data steward to outline appropriate data management by the researcher, including possible consequences where those expectations are not met. Those who abstained agreed with the spirit of the recommendation but argued that the data access contracts should be made once access is approved, not at the time of application.

Finally, participants expressed a desire for those using linked data to be trained adequately in a certificate program to avoid inappropriate use and security issues. Those who abstained were concerned that it was not enough for data users to be trained and that there needed to be a separate auditing process to ensure users’ compliance with regulations. Those who were against believed the researcher training was redundant because data stewards are already vetting researchers.

### 4. Involvement of the public

Participants were interested in transparency around what data are shared and with whom, indicating it was important simply for the public to be aware of these issues. They suggested this transparency could be satisfied by a website listing the data access requests, showing the approvals, denials, and reasons for the decisions. Some participants commented that the website did not need to extend to denied requests. Those who abstained from voting on public disclosure (statement 18), agreed in principle with the contents but felt there would also be potential for misinterpretation, and that the disclosure itself could raise concerns (e.g., a denied research request may cause unfounded public alarm). Those voting against potentially alarmist public disclosure said such disclosure was not useful because the public would be largely disinterested in the data access requests and would not seek them out.

**Table:** 

Statements and recommendations – Researcher responsibilities	Y	N	A

15. Researchers have some responsibility to vulnerable populations they study or identify as vulnerable in their research.	23	0	0
16. Anyone seeking access to linked data must sign a standardized contract outlining confidentiality requirements and further dissemination of data.	19	0	4
17. Data security certificate program should be established and it should be mandatory for people who are using linked data.	18	3	2

**Table:** 

Statements – Involvement of the public	Y	N	A

18. There should be public disclosure (e.g. on a website) of requests for access to data. This should include approvals, denials, and reasons for those decisions.	14	2	7
19. Transparency and disclosure of research requests is sufficient as a form of public consultation	16	1	6

Finally, there was discussion of whether or how the public should be involved in the review of data access requests. Statement 19 reaffirmed that transparency was adequate and no other form of public consultation was necessary. Those who abstained from voting on this statement were concerned that the term “transparency” was too vague. Both those who abstained and those who voted against the statement felt that mere disclosure and transparency would set too low of a bar for government accountability when engaging with the public.

## Discussion

This public deliberation provided further evidence that an informed and diverse mini-public is able to develop nuanced policy-relevant recommendations [17] and was supportive of research using linked data because of the value it can provide to society. This is consistent with public sentiment found in research conducted in other jurisdictions [6,7,34,35]. Many of the participants’ recommendations matched current best-practices in data sharing in BC; however, they were the result of careful consideration by participants and not merely their acceptance of current practices. Participants expressed a desire to see that the data access request process be made more efficient to facilitate more research, as long as there are adequate provisions for security and privacy. They developed a number of policy-relevant statements that addressed infrastructure, practice guidelines, public engagement, and training for both data stewards and researchers. The statements were presented to knowledge-users both in the final deliberative session, in a document summarizing the results, and in a range of presentations. Diverse knowledge-users, including data stewards and policy makers, now have formally documented public input that they were seeking to support policy and practice decisions.

### Relationship to current practices

Many of the statements that the participants developed reflected current data sharing practices that are common across jurisdictions. For example, participants made statements about researchers signing data access contracts, and requiring both ethics and peer review, all of which are part of current practice [36] and part of the “five safes” framework that is gaining international traction [37]. They also recognized that reviewing data access requests was currently done by data stewards and approved of this role, though they thought that training and procedures would benefit from standardization and the codification of practices for data stewards and researchers.

In some cases, participants abstained or voted against statements that supported current practices (e.g., statement 9, research reviews by ethics boards). They did so because they believed that those practices are currently in use; this was despite being asked to vote without taking account of current practices. The description of current practices is something that future deliberations may wish to consider and perhaps address more explicitly in training materials. Participants were also wary of creating additional systems that would slow the data access process and decrease efficiency. For example, this was the reasoning offered for not supporting additional ethics reviews or certification bodies for data stewards. Hence, participants’ lack of support for some statements should not be taken as a lack of support for the proposed statement itself, but rather an expression of their strong desire for efficiency.

### Future directions

A number of the participants’ statements offer opportunities for changes in data sharing practices. The most significant changes have to do with standardization both in terms of the data access processes and guidelines as well as training, particularly for data stewards. It is also important to note what aspects of current governance participants thought were important so that these are not lost in subsequent policy changes.

There is no existing research literature on data steward practices and no evidence of any jurisdiction with clear standards and/or training for data stewards. Currently, data stewards in British Columbia do not follow common processes and guidelines and can differ widely in terms of how and how quickly they may process data access requests. While there is legislation that generally refers to how personal information should be shared for research (*British Columbia Freedom of Information and Protection of Privacy Act* [38]), the interpretation of the legislation is not uniform and can vary between individuals and institutions. These differences can lead to disagreements between data stewards when working with data sets that are intended to be linked, which in turn may lead to longer waiting times for approvals and occasionally cancelled data access requests by researchers when data becomes irrelevant to their projects.

Increasing standardization was something participants felt could improve not only efficiency, but also data security (i.e., by establishing guidelines for data storage). To achieve this, some participants suggested the creation of a certifying body for data stewards that would be responsible for training and guidelines. Yet, this suggestion did not receive unanimous support, because, as mentioned above, participants were concerned that the additional requirements would introduce new inefficiencies. In a similar vein, participants frequently commented on the need for a risk-based approach for efficient decision making (e.g., proportionate governance [36,39]). They did not produce a recommendation on this because they believed such an approach was already in place.

Participants spent some time talking about transparency, which they conceived in general terms, such as having a public listing of existing data access requests or research currently underway. While a form of these listings exists in some organizations, most data managing organizations do not practice this level of transparency [40,41]. Participants suggested that making this available, possibly through a website, could improve the trustworthiness of the data access process. Aside from the statement about transparency, they did not make specific recommendations about how the public should be involved in the decision-making process. With some exceptions, participants in the deliberation were on the whole not convinced that greater or deeper public involvement was required on the issues discussed in this deliberation. This sentiment is reflected in Statements 18 and 19. Indeed, many of the participants seemed to take a somewhat fatalistic approach to questions relating to their personal data, such that they felt that there were certain controls over information they could not have in a modern society. We appreciate the irony of this particular outcome, given that the deliberative process in which the participants formulated their recommendation was precisely such a mechanism for deeper public involvement in these issues. We are not sure how much this sentiment reflects broader public opinion, or whether it is a result of the particular sample or contingencies such as the information provided to the participants. We intend to investigate the issue further in a follow-up project.

### Future deliberations

There were some statements on which participants did not reach consensus, and which could provide opportunities for future investigation. In accordance with previous deliberative engagements on data sharing [7,35], the participants diverged on how private enterprises, specifically corporations, should be involved in research. While participants were wary of the intentions of corporations in conducting research (i.e., profit motives), many considered that their involvement was inevitable. There was discussion on limiting corporations’ direct involvement in the research, but this was tempered by an acknowledgement that it was unlikely corporations would invest in research without the opportunity to benefit from it. Further, there was a concern that if corporations were constrained they would no longer collaborate with researchers. We are actively pursuing the question of corporate involvement for a future planned deliberation.

Finally, while participants indicated that transparency was an important aspect of involving the public in data access, they said it was not sufficient. Further deliberations could delve more deeply into both the meaning and enactment of transparency around the use of linked data for research.

## Conclusion

While members of the public are concerned about the privacy and security of their data, they are also concerned about data access policies that limit the use of linked data for research that has public value. This public deliberation adds further evidence that the public strongly recognizes the benefit of research and wishes research to be supported. The statements crafted by this mini-public articulate some features of a secure, standardized and transparent framework for research data access. Policies should respond to public interests by improving the efficiency of data sharing through standardization and transparency, which would facilitate research and security while building trustworthiness with people who are represented in those data.

## Ethics approval and consent to participate

This study was approved by the University of British Columbia Research Ethics Board (H17-03500). All participants signed a written informed consent form prior to the event.

## Abbreviations

Canadian Institutes of Health Research (CIHR)

University of British Columbia (UBC)

## Supplementary Appendix 1: description of the demographic characteristics of the deliberation participants

Sex:

Male:	16
Female:	12

Age:

18-24:	4
25-34:	7
35-49:	6
50-64:	7
65+:	4

Household income:

$20,000-$34,999:	4
$35,000-$49,999:	3
$50,000-$79,999:	9
$80,000+:	12

Ethnic identity:

African American:	1
White:	15
Chinese:	7
Egyptian:	1
Metis:	1
South Asian:	3

Geographic location by Provincial Health Authority (a map of BC Health authorities can be found here: https://www2.gov.bc.ca/gov/content/health/about-bc-s-health-care-system/partners/health-authorities/regional-health-authorities

Vancouver Coastal:	6
Fraser Valley:	7
Vancouver Island:	7
Northern BC:	4
Interior BC:	4

Metropolitan influence zone [23]: (higher numbers indicate cities that are further away from metropolitan areas)

MIZ 1:	19
MIZ 2:	5
MIZ 3:	3
MIZ 6:	1
